# Whole genome sequencing reveals genomic heterogeneity and antibiotic purification in *Mycobacterium tuberculosis* isolates

**DOI:** 10.1186/s12864-015-2067-2

**Published:** 2015-10-24

**Authors:** PA Black, M. de Vos, GE Louw, RG van der Merwe, A. Dippenaar, EM Streicher, AM Abdallah, SL Sampson, TC Victor, T. Dolby, JA Simpson, PD van Helden, RM Warren, A. Pain

**Affiliations:** DST-NRF Centre of Excellence for Biomedical Tuberculosis Research/SA MRC Centre for Tuberculosis Research, Division of Molecular Biology and Human Genetics, Faculty of Medicine and Health Sciences, Stellenbosch University, Tygerberg, South Africa; Pathogen Genomics Laboratory, BESE Division, King Abdullah University of Science and Technology, Thuwal, Saudi Arabia; National Health Laboratory Services, Green Point, Cape Town, South Africa

**Keywords:** Genetic complexity, Clinical isolates, *Mycobacterium tuberculosis*, Heterogeneity, Next generation sequencing, Relaxed variant filtering

## Abstract

**Background:**

Whole genome sequencing has revolutionised the interrogation of mycobacterial genomes. Recent studies have reported conflicting findings on the genomic stability of *Mycobacterium tuberculosis* during the evolution of drug resistance. In an age where whole genome sequencing is increasingly relied upon for defining the structure of bacterial genomes, it is important to investigate the reliability of next generation sequencing to identify clonal variants present in a minor percentage of the population. This study aimed to define a reliable cut-off for identification of low frequency sequence variants and to subsequently investigate genetic heterogeneity and the evolution of drug resistance in *M. tuberculosis. *

**Methods:**

Genomic DNA was isolated from single colonies from 14 rifampicin mono-resistant *M. tuberculosis* isolates, as well as the primary cultures and follow up MDR cultures from two of these patients. The whole genomes of the *M. tuberculosis *isolates were sequenced using either the Illumina MiSeq or Illumina HiSeq platforms. Sequences were analysed with an in-house pipeline.

**Results:**

Using next-generation sequencing in combination with Sanger sequencing and statistical analysis we defined a read frequency cut-off of 30 % to identify low frequency *M. tuberculosis* variants with high confidence. Using this cut-off we demonstrated a high rate of genetic diversity between single colonies isolated from one population, showing that by using the current sequencing technology, single colonies are not a true reflection of the genetic diversity within a whole population and vice versa. We further showed that numerous heterogeneous variants emerge and then disappear during the evolution of isoniazid resistance within individual patients. Our findings allowed us to formulate a model for the selective bottleneck which occurs during the course of infection, acting as a genomic purification event.

**Conclusions:**

Our study demonstrated true levels of genetic diversity within an *M. tuberculosis* population and showed that genetic diversity may be re-defined when a selective pressure, such as drug exposure, is imposed on *M. tuberculosis* populations during the course of infection. This suggests that the genome of *M. tuberculosis* is more dynamic than previously thought, suggesting preparedness to respond to a changing environment.

**Electronic supplementary material:**

The online version of this article (doi:10.1186/s12864-015-2067-2) contains supplementary material, which is available to authorized users.

## Background

Whole genome sequencing (WGS) has revolutionised the detailed interrogation of mycobacterial genomes at base-pair resolution. Application of this technology has provided novel insights into the evolution of members of the *Mycobacterium tuberculosis* complex [1–3]. More recently, WGS has been applied to investigate the evolution of drug resistance based on the hypothesis that additional mutational events may precede or occur concurrently with known resistance conferring mutations [4–8]. A number of recent studies have assessed the genomic stability of *M. tuberculosis* during the evolution of drug resistance (Reviewed by Trauner et al., 2014) [4], producing diametrically opposed results. Some studies have demonstrated genomic stability [6, 7, 9, 10], while more recent reports have shown genomic instability with the emergence of additional genetic variants independent of those observed in drug target genes conferring resistance [5, 11–14]. Various studies have demonstrated the acquisition of known resistance conferring mutations in the *M. tuberculosis* genome during the course of infection and subsequent drug treatment. This highlights the potential of *M. tuberculosis* to diversify and adapt under selective pressure [11, 13, 15]. In other work, WGS analysis of serially collected sputum samples of Tuberculosis (TB) patients provided overwhelming evidence of the presence of drug resistant sub-populations [5]. This study emphasised the need to investigate the implications of inter- and intra-host *M. tuberculosis* genetic diversity for transmission and disease outcomes [5]. The presence of drug resistant sub-populations is a major concern when considering the variable sensitivity of standard genetic and microbiological tests for the diagnosis of drug resistance *M. tuberculosis* [16].

It is important to note that the identification of sub-populations within the context of WGS is dependent on the read frequency cut-off values used in the standard variant filtering approach. Typically, these frequency cut-off values are set at >70 %, implying that only variants fixed within a population are identified i.e. variants present at only >70 % of the sequencing reads are analysed [6, 8]. Failure to adjust these algorithms to accommodate for the presence of low-frequency sub-populations has led authors to conclude that the population structure of *M. tuberculosis* is homogeneous [6, 8, 17, 18]. By using an alternative variant calling approach (a minimum read depth of 50 and a minimum variant frequency of 4 %) a recent study demonstrated the rapid expansion and collapse of different sub-populations that evolved in parallel during the evolution of extensively drug resistant (XDR-TB) [14]. However, while WGS is increasingly being used to investigate drug resistance and evolution in *M. tuberculosis,* there is still uncertainty surrounding the error rate of sequencing, read alignment and the detection of variants. In an age where WGS is increasingly relied on for defining the structure of bacterial genomes, it is important to investigate the reliability of next-generation sequencing reads where a variant is only present in a minor percentage of the sequencing reads. This therefore questions the threshold at which underlying populations, as indicated by the percentage of sequencing reads supporting a variant allele, can be confirmed as true variants as opposed to sequencing errors.

This study aimed to define a reliable cut-off for identification of heterogeneous variants from WGS data. Subsequently this cut-off was used to investigate: 1) heterogeneity in single colonies isolated from 13 rifampicin mono-resistant *M. tuberculosis* clinical isolates, and 2) the evolution of isoniazid resistance in rifampicin mono-resistant isolates in 2 patients.

## Results

Strains were initially selected on the basis of clinical diagnostic records, then subjected to further analysis to confirm their resistance profiles and strain genotypes. Phenotypic drug resistance testing of the parental isolates (*n* = 13) and their associated single colonies (*n* = 36) confirmed the rifampicin mono-resistant profile in all cases. As expected, all isolates carried a mutation in the *rpoB* gene; genotyping of parental isolates identified one of the following mutations in the *rpoB* gene: Ser531Leu, His526Tyr or Leu533Pro (Table 1). These mutations were retained in the corresponding single colonies after selection on agar plates containing rifampicin. IS*6110* genotyping and spoligotyping revealed that the rifampicin mono-resistant isolates originated from different genetic backgrounds (LCC, Haarlem, Beijing and EAI), representing the broad strain diversity circulating in the Western Cape, South Africa. Genotyping by IS*6110* also showed that the parental isolates and single colonies were identical, confirming the absence of mixed infection.

**Table 1:** Genotypic characterisation of *M. tuberculosis* clinical isolates used for the investigation into genomic heterogeneity

Isolate name	rpoB^a^	Spoligotype classification
R160	Ser531Leu	LCC
R376	Ser531Leu	Haarlem
R458	Ser531Leu	Unknown/unique
R486	Leu533Pro	Beijing
R631	His526Tyr	Unknown/unique
R637	Ser531Leu	Beijing
R641	Leu533Pro	Beijing
R721	Ser531Leu	Beijing
R912	His526Tyr	EAI
R965	Leu533Pro	Beijing
R966	His526Tyr	Beijing
R1035	Ser531Leu	LAM
R1415	His526Tyr	Beijing

*LCC* low copy clade, *EAI* East African Indian, *LAM* Latin American Mediterranean

^a^Amino acid change according to the *Escherichia coli rpoB* gene sequence

### Identifying a reliable cut-off to detect genomic heterogeneity

To define a reliable cut-off for heterogeneous variant detection, we employed a combination of WGS and statistical analyses of parental isolates and their associated single colonies. Single colonies were obtained by plating serial dilutions of the parental isolate onto 7H10 agar containing 2 μg/ml RIF. Two to three single colonies isolated from each of the 13 parental rifampicin mono-resistant *M. tuberculosis* isolates (Table 1) were selected and subjected to WGS analysis. This analysis of 36 single colonies revealed the presence of a total of 153 possible sequence variants between corresponding single colonies using *M. tuberculosis* H37Rv as the alignment standard. To determine whether a variant which was present in less than 100 % of the reads reflected the presence of either genetically distinct subpopulations or sequencing artefacts, 46 of the 153 possible variants were selected for validation by Sanger sequencing (Table 2). All 6 variants present in ≥71.8 % of the reads were verified with a single chromatogram peak demonstrating that only the dominant nucleotide was detected. In addition, 25 variants present in between 30.5 and 70.7 % of the reads were also determined to contain two nucleotides indicated by the presence of two chromatogram peaks. Of the 16 variants with a read frequency between 20.3 and 30.5 %, 7 were shown to be false positives as only a single chromatogram peak was observed. We investigated the frequency of the 7 false positive variants across all of the single colonies as recurrence of these variants in other genomes might suggest that the false variants were called due to mapping errors. Only one of the 7 variants (in *Rv0282*) was found to be recurrent and identified in 22 of the single colonies.

**Table 2:** Validation of variants with a read frequency ranging between 20 and 100 % using targeted PCR and sanger sequencing

Gene^a^	Sanger chromatogram result	Read frequency (%)	Sanger result
*Rv2316*	Single peak	127/127 (100.0)	True
*Rv1703c*	Single peak	152/154 (98.7)	True
*Rv0820*	Single peak	193/200 (96.5)	True
*Rv3220c*	Single peak	129/134 (96.3)	True
*Rv0537c*	Single peak	98/126 (77.8)	True
*Rv2692*	Single peak	102/142 (71.8)	True
*Rv1521*	Double peaks	157/222 (70.7)	True
*Rv0521*	Double peaks	117/159 (68.8)	True
*Rv1904*	Double peaks	119/181 (65.7)	True
*Rv1230c*	Double peaks	96/155 (61.9)	True
*Rv3086*	Double peaks	84/145 (57.9)	True
*Rv3083*	Double peaks	55/95 (57.9)	True
*Rv1429*	Double peaks	74/129 (57.4)	True
*Rv2577*	Double peaks	33/61 (54.1)	True
Intergenic (1093238)	Double peaks	89/168 (53.0)	True
*Rv3391*	Double peaks	57/113 (50.4)	True
*Rv2689c*	Double peaks	59/123 (48.0)	True
*Rv0970*	Double peaks	48/104 (46.2)	True
*Rv2173*	Double peaks	54/130 (41.5)	True
*Rv1929c*	Double peaks	52/128 (40.6)	True
*Rv2459*	Double peaks	18/45 (40.0)	True
*Rv2984*	Double peaks	34/89 (38.2)	True
*Rv1894c*	Double peaks	36/95 (37.9)	True
*Rv1316c*	Double peaks	72/198 (36.4)	True
*Rv1021*	Double peaks	42/119 (35.3)	True
*Rv2544*	Double peaks	50/151 (33.1)	True
*Rv3772*	Double peaks	47/147 (32.7)	True
*Rv3861*	Double peaks	38/119 (31.9)	True
*Rv3780*	Double peaks	30/106 (31.9)	True
*Rv0491*	Double peaks	44/138 (31.9)	True
*Rv2957*	Single peak	47/154 (30.5)	False
*Rv1549*	Double peaks	42/138 (30.4)	True
*Rv3703c*	Single peak	37/126 (29.4)	False
*Rv0594*	Double peaks	61/202 (29.2)	True
*Rv0372c*	Single peak	85/296 (28.7)	False
*Rv1479*	Single peak	34/120 (28.3)	False
*Rv0780*	Single peak	44/159 (27.7)	False
*Rv0092*	Double peaks	47/170 (27.6)	True
*Rv0688*	Double peaks	56/209 (26.8)	True
*Rv0282*	Single peak	54/205 (26.3)	False
*Rv0663*	Double peaks	41/158 (25.9)	True
*Rv0522*	Double peaks	66/263 (25.1)	True
*Rv1660*	Double peaks	52/239 (21.8)	True
*Rv1627c*	Single peak	34/161 (21.1)	False
*Rv0654*	Double peaks	34/167 (20.4)	True
*Rv2934*	Double peaks	47/232 (20.3)	True

^a^All variant positions and WGS results are listed in the supplementary data (Additional file 2: Table S2)

**Fig. 1:**
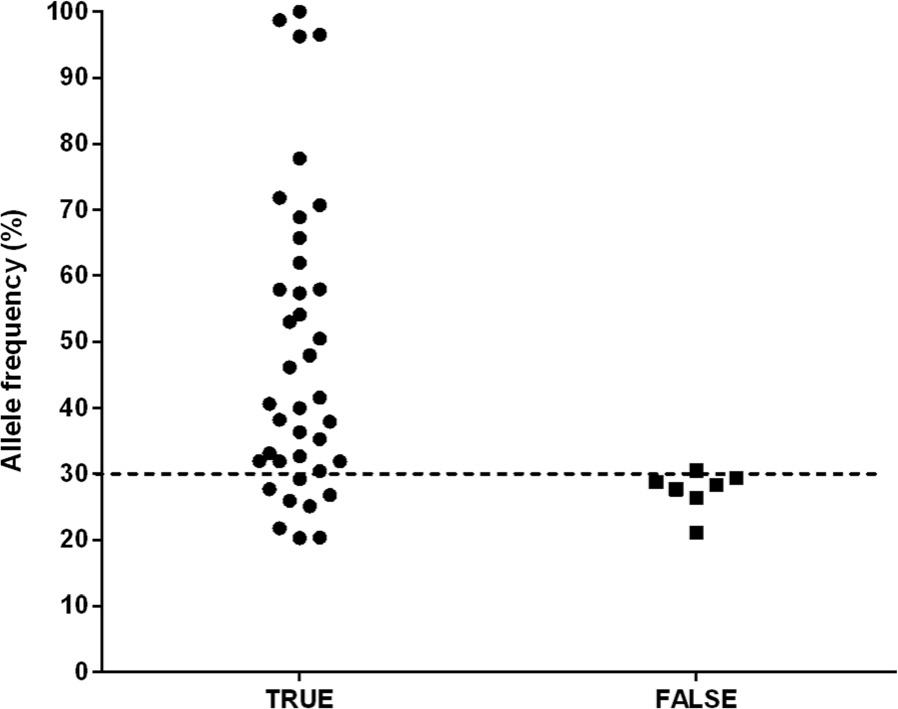
Validation of variants identified by Illumina sequencing. Analysis of Sanger sequencing alignments and corresponding chromatograms was used to validate the presence of homo- and heterogeneous variants identified by Illumina sequencing. Variants present in either the sequencing file or in the chromatogram (Additional file 2: Table S2) were scored as true variants while sequences which remained wild-type were scored as false

We performed a receiver operator characteristic (ROC) curve analysis to define a reliable cut-off for the identification of true heterogeneous variants. ROC curve analysis disproved the null hypothesis, with an area under the curve (AUC) of 0.835 (95 % CI: 0.722 – 0.948). A cut-off value of 30 % read frequency was defined with a true positive value of 0.795 and a false positive value of 0.143 i.e. a true positive rate of 79.5 % and a false positive rate of 14.3 % (Fig. 1). Using the conventional cut-off value of 70 % there would be a true positive rate of 17.9 % and a false positive rate of 0 %. Accordingly, we defined a cut off for the description of true heterogeneity at a defined nucleotide position as ≥30 % i.e. if a variant identified in at least 30 % of the Illumina sequencing reads (after filtering and GenomeView analysis), it is likely that the variant is truly present within the genome of a sub-population of bacilli. Using this analysis we also defined a variant with a read frequency of greater than 70 % to be fixed within a population.

### Identifying genomic heterogeneity in individual colonies from rifampicin mono-resistant isolates

We next applied the ≥30 % variant frequency cut-off value to assess genomic heterogeneity within and between individual colonies. Using this cut-off value this analysis 36 of single colonies revealed the presence of a total of 114 possible sequence variants between corresponding single colonies using *M. tuberculosis* H37Rv as the alignment standard. From the 114 possible variants, 42 were found to have a read frequency of greater than 70 %, while 72 were found to have a read frequency of between 30 and 70 % (Table 3, (Additional file 1)). The number of variants identified was independent of the strain background, and ranged from 0 to 19 in different clinical isolates. Importantly, genomic heterogeneity was observed both within single colonies (where a variant is only present in a proportion of the reads) and between single colonies isolated from one parent.

**Table 3:** Variants identified in corresponding single colonies derived from different clinical isolates

	Single colony 1			Single colony 2			Single colony 3			
	Total^a^	Fixed^b^	Hetero^c^	Total^a^	Fixed^b^	Hetero^c^	Total^a^	Fixed^b^	Hetero^c^	Total variation
R160	5	2	3	4	3	1	-	-	-	9
R376	3	1	2	8	2	6	6	1	5	17
R458	3	3	0	5	2	3	3	0	3	11
R486	0	0	0	2	0	2	-	-	-	2
R631	5	0	5	6	0	6	8	0	8	19
R637	0	0	0	0	0	0	0	0	0	0
R641	9	9	0	3	0	3	6	0	6	18
R721	0	0	0	4	0	4	0	0	0	4
R912	4	4	0	6	3	3	7	7	0	17
R965	4	4	0	1	1	0	-	-	-	5
R966	5	0	5	1	0	1	6	0	6	12
R1035	0	0	0	0	0	0	0	0	0	0
R1415	0	0	0	0	0	0	0	0	0	0

^a^Total number variants unique between the corresponding single colonies

^b^Fixed variants as defined as having a read frequency of ≥ 70 %

^c^Heterogeneous variants as defined as having a read frequency < 70 % and ≥ 30 %

-A third single colony was not available for the comparison

### Comparing single colonies and the entire population isolated from sputum

**Fig. 2:**
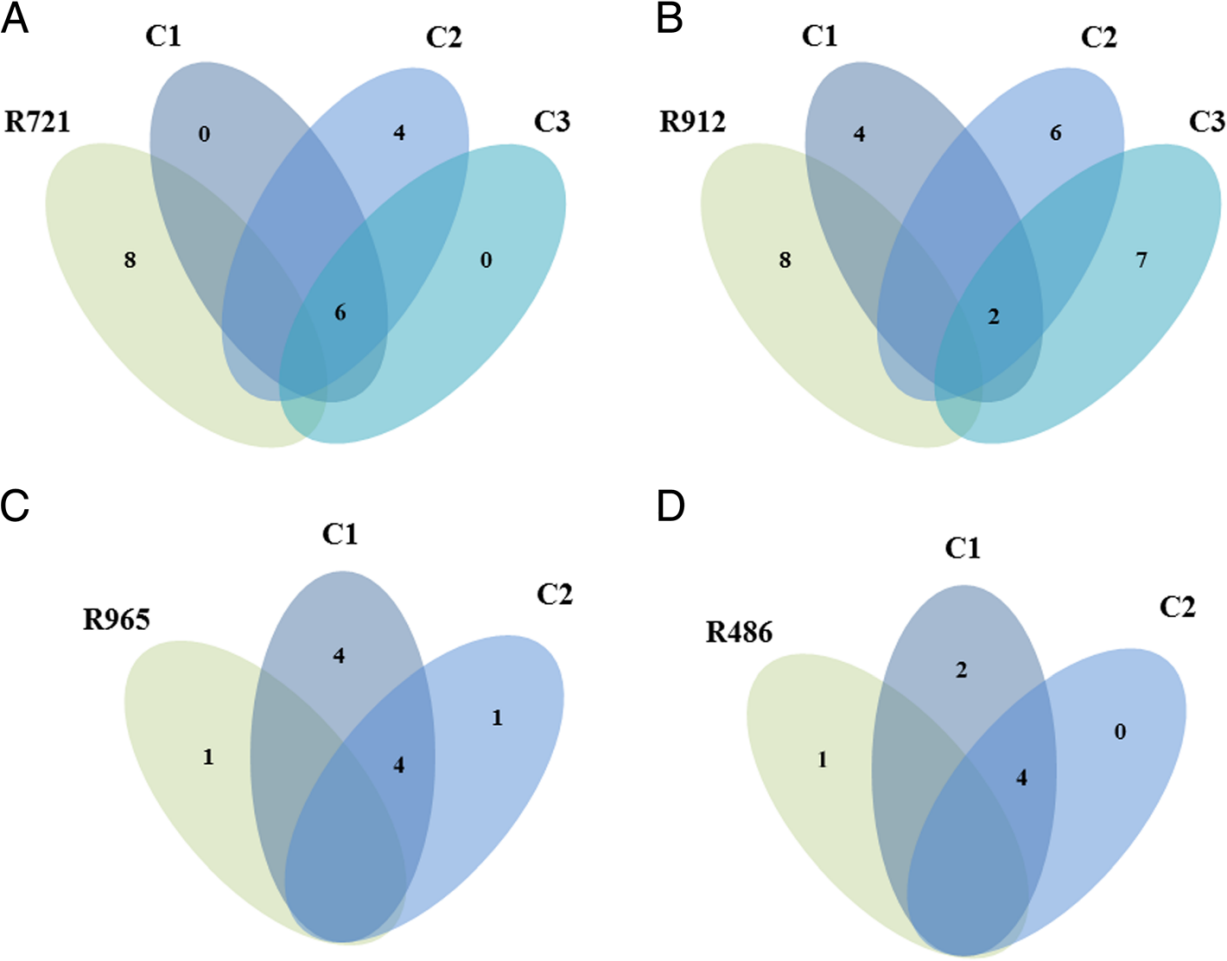
Genetic diversity between parental populations and their corresponding single colonies. **a** The R721 parental population had 8 unique variants relative to all three colonies. While all three colonies shared 6 variants which were unique to the colonies relative to the parental population, each colony also harboured unique variants relative to the other colonies. Similarly, for (**b**) the R912 parental population had 8 unique variants relative to all three colonies. While all three colonies only shared 2 variants, each colony also harboured unique variants relative to the other colonies. (**c**) For R965, the parental population had 1 unique variant relative to both colonies, while the colonies shared 4 variants which were unique relative to the parental population. Similarly, the two colonies for R486 (**d**) shared 4 variants which were unique relative to the parental population. The R486 parental population had 1 unique variant relative to the other colonies

WGS data was available for four of the parental *M. tuberculosis* isolates used to make the single colonies. Therefore, to determine whether the single colonies reflected the genetic heterogeneity of the parent isolate, we compared the WGS of the single colonies from four rifampicin mono-resistant *M. tuberculosis* clinical isolates to the sequences of their corresponding parent population (R721, R912, R965 and R486). As shown in Fig. 2 this comparison identified 6, 4, 4 and 2 variants that were unique to the single colonies relative to the parental genomes for R721, R965, R486 and R912, respectively. These variants were shared between all of the single colonies cultured from their corresponding parental isolates. In addition, the individual single colonies also harboured unique variants that were absent in the corresponding parental isolates as well as absent in the other related single colonies. Conversely, the parental isolates also each harboured unique variants that were absent in all corresponding single colonies (Fig. 2). Together these results suggest that all of the genomic variants identified as unique to the single colonies were masked by the dominant population present in the parent isolates.

### Intra-patient evolution of drug resistance

Having established the utility of our analytical approach in identifying inter- and intra-isolate variation, we next went on to analyse genome heterogeneity during the evolution of drug resistance. Here, we analysed MDR isolates (R807 and R1210) collected approximately 7 and 11 months after the rifampicin mono-resistant isolates R721 and R912, respectively (Table 4). We investigated the heterogeneity of SNPs across the genome of the rifampicin mono-resistant and MDR entire populations relative to the *M. tuberculosis* H37Rv reference genome. We subsequently compared this list of SNPs between the rifampicin mono-resistant and MDR isolates to identify variants unique to each isolate as well as the evolutionary events associated with the emergence of drug (isoniazid) resistance (Table 5).

**Table 4:** *M. tuberculosis* clinical isolates demonstrating a number of unique variants between rifampicin mono-resistant and MDR isolates during *in vivo* evolution of isoniazid resistance

Patient	Isolate name	Phenotypic resistance	Collection date	rpoB^c^	*katG*	*inhA* promoter	Spoligotype classification	Fixed variants^a^	Heterogeneous variants^b^	Total variation
**1**	R721	Rifampicin mono	22/10/2003	Ser531Leu	-	-	Beijing	1	8	9
	R807	MDR	19/05/2004	Ser531Leu	Gly309Val	-	Beijing	2	0	2
**2**	R912	Rifampicin mono	15/09/2004	His526Tyr	-	-	East Africa Indian (EAI)	7	0	7
	R1210	MDR	12/08/2005	His526Tyr	-	−15	EAI	1	5	6

^a^Fixed variants as defined as having an read coverage of ≥70 %

^b^Heterogeneous variants as defined as having an read coverage <70 % and ≥30 %

^c^Amino acid change according to the *Escherichia coli rpoB* gene sequence

**Table 5:** Isolate specific variants identified in rifampicin mono-resistant and MDR *M. tuberculosis* isolates of patient 1 and 2

	Isolate	Locus	*Gene*	Amino acid change	Coverage of variant (%)	Gene description	Functional category^C^
Patient 1	R721^a^	Rv0435c		I397L	50	Putative conserved ATPase	Cell wall and cell processes
		Rv0435c		D395Y	50	Putative conserved ATPase	
		Rv0668	*rpoC*	V1039A	30	DNA-directed RNA polymerase RpoC (RNA polymerase beta’ subunit).	Information pathways
		Rv1850	*ureC*	Q11K	48	Urease alpha subunit UreC (urea amidohydrolase)	Intermediary metabolism and respiration
		Rv1850	*ureC*	Q11R	49	Urease alpha subunit UreC (urea amidohydrolase)	
		Rv3218		Y174H	66	Conserved protein	Conserved hypotheticals
		Rv2004c		Ins AAG	43	Conserved protein	Conserved hypotheticals
		Rv3563	*fadE32*	Ins AC	40	Probable acyl-CoA dehydrogenase FadE32	Lipid metabolism
		Rv3696c	*glpK*	Ins AC	73	Probable glycerol kinase GlpK (ATP:glycerol 3-phosphotransferase)	Intermediary metabolism and respiration
	R807^b^	Rv1908c	*katG*	G309V	100	Catalase-peroxidase-peroxynitritase T KatG	Virulence, detoxification, adaptation
		Rv3696c	*glpK*	T91I	80	Probable glycerol kinase GlpK (glycerokinase) (GK)	Intermediary metabolism and respiration
Patient 2	R912^a^	Rv1128c		G430S	98	Conserved hypothetical protein	Insertion sequences and phages
		Rv2236c	*cobD*	L269S	97	Probable cobalamin biosynthesis transmembrane protein CobD	Intermediary metabolism and respiration
		Rv2664		H22Q	99	Hypothetical protein	Conserved hypotheticals
		Rv2772c		E149*	97	Probable conserved transmembrane protein	Cell wall and cell processes
		Rv2984	*ppk1*	P631A	96	Polyphosphate kinase PPK (polyphosphoric acid kinase)	Intermediary metabolism and respiration
		Rv3391	*acrA1*	syn (248)	99	Possible multi-functional enzyme with acyl-CoA-reductase activity AcrA1	Lipid metabolism
		Rv3537	*kstD*	syn (378)	98	Probable dehydrogenase	Intermediary metabolism and respiration
	R1210^b^		*inhA promoter*	−15	45		
		Rv1484	*inhA*	S94A	70	NADH-dependent enoyl-[acyl-carrier-protein] reductase InhA (NADH-dependent enoyl-ACP reductase)	Lipid metabolism
		Rv1629	*polA*	syn (146)	45	Probable DNA polymerase I PolA	Information pathways
		Rv2935	*ppsE*	C582R	69	Phenolpthiocerol synthesis type-I polyketide synthase PpsE	Lipid metabolism

^a^Rifampicin mono-resistant

^b^MDR

^c^Functional category as classified by Tuberculist (http://genolist.pasteur.fr/TubercuList/ and http://tuberculist.epfl.ch/)

**Fig. 3:**
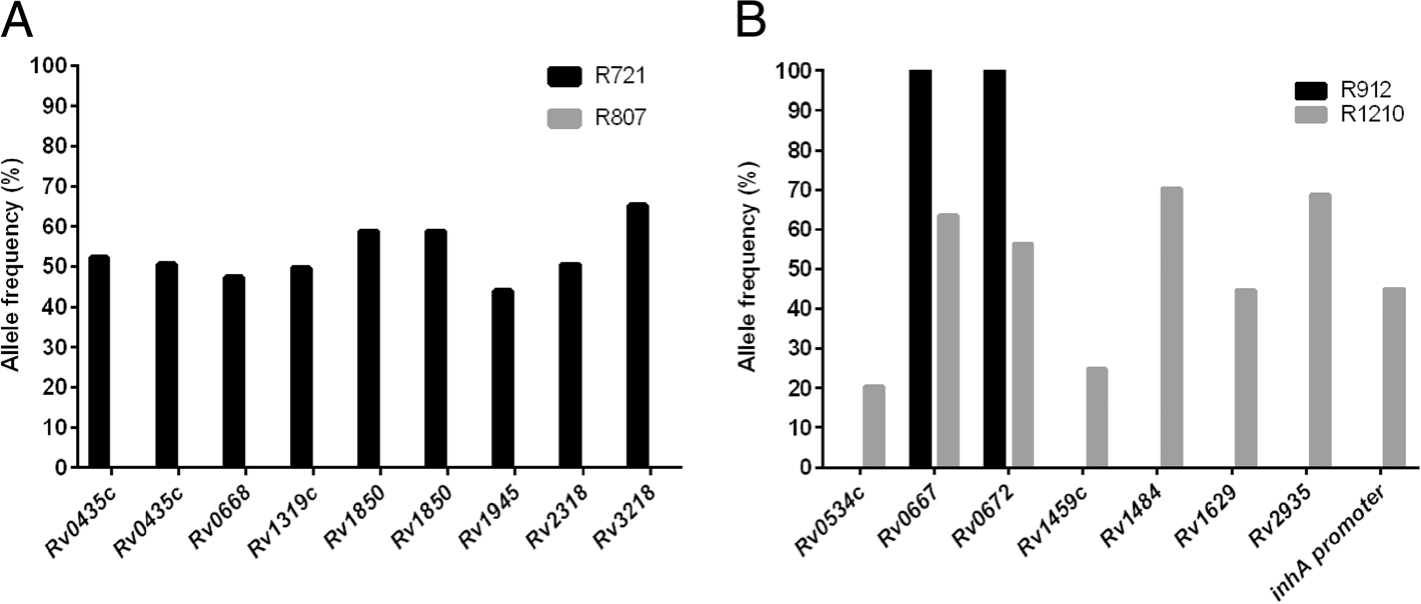
Heterogeneous positions identified across the whole population genomes relative to *M. tuberculosis* H37Rv reference genome. **a**The rifampicin mono-resistant isolate (R721) for patient 1 shows numerous heterogeneous variants relative to *M. tuberculosis* H37Rv while the follow-up MDR isolate (R807) has none. (**b**) For patient 2 the rifampicin mono-resist isolate (R912) showed no heterogeneous variants relative to *M. tuberculosis* H37Rv, while the follow-up MDR isolate (R1210) had numerous heterogeneous variants. R912 shared 2 variants (Rv0667 and Rv0672) with R1210, where the variant was present at 100 % in R912 but was a heterogeneous variant in R1210

For patient 1 numerous heterogeneous variants were identified in the rifampicin mono-resistant isolate, R721, while there were no heterogeneous variants observed in the follow-up MDR isolate, R807 (Fig. 3a, Table 4). Since the primary difference between these two isolates is the presence of a *katG* mutation (Table 5), these results suggest that the heterogeneous variants present in R721 were ‘lost’ during the acquisition of isoniazid resistance. Patient 2 shows contrasting results: the rifampicin mono-resistant isolate, R912, was shown to have no heterogeneous variants while its follow-up MDR isolate, R1210, harboured 5 (Fig. 3b). The variants shown to be unique to R912 were all present at a variant frequency of greater than 70 %. Interestingly, two variants shown to be fixed within the rifampicin mono-resistant population, with a variant frequency of 100 %, were found to be present at a lower frequency within the MDR population (Fig. 3b). This finding indicates that two fixed variants in R912 may have been lost in R1210. These results revealed the acquisition and loss of numerous heterogeneous variants (Table 5), suggesting continuous genome evolution despite the evolutionary bottle neck imposed by the isoniazid selective pressure.

Finally, we compared the WGS data of MDR isolates to the single colonies generated from the rifampicin mono-resistant parental isolates. This comparison failed to identify any of the single colony specific genomic variants in the MDR isolate.

## Discussion

Our study aimed to use WGS in combination with Sanger sequencing and statistical analyses to define a reliable cut-off for heterogeneous variant detection. Our data enabled us to define a read frequency cut off of 30 % for reliable Illumina sequencing variant frequency filtering i.e. a variant present in 30 % or more of the sequencing reads can be considered to be a true variant and not a sequencing error. At a variant frequency of 30 % there is a true positive of 79.5 % and a false positive rate of 14.3 %. We acknowledge that there is still a chance that true variants at a frequency lower than 30 % may be missed due to the limited resolution of Sanger sequencing. For the purpose of our analyses we regard it of greater importance to exclude false positives from our analyses than to omit a small amount of true positives. However, we do not exclude the probability that this cut-off value may change with increased sequence read depth. Higher depth of coverage would however not limit the detection of false positive variants as the 7 false positive variants identified in this study were not associated with lower mean genome coverage. Furthermore, by defining a cut-off it allowed us to use a relaxed variant filtering approach to investigate the presence of sub-populations in *M. tuberculosis* clinical isolates.

Using our cut-off of 30 % variant frequency we investigated the genomic heterogeneity within and between individual single colonies isolated from rifampicin mono-resistant *M. tuberculosis* clinical isolates. To our knowledge this is the first study that has investigated *M. tuberculosis* genomic diversity within single colonies from a clinical sample at a single time point. We observed a high rate of genetic diversity between single colonies isolated from the same parental isolate. We acknowledge that the analysis of only two to three single colonies for each patient isolate would have limited the anlaysis of the true heterogeneity in the total population. We also acknowledge that the selection of these single colonies on media containing rifampicin would have resulted in the loss of variants representing rifampicin susceptible colonies. This high rate of genetic diversity seen in our study is in concordance with previous studies that used relaxed filtering with regards to variant frequency [5, 12, 14]. Sun et al*.* showed that there can be as many as 41 (ranging between 6 and 41) variants between serial sputum samples, with 82.7 % of all the variants at frequencies lower than 20 % (based on statistical evaluation as opposed to validation by Sanger sequencing) [5]. A study by Bryant et al*.* showed seemingly contrasting results using the same filtering approach to investigate relapse and reinfection cases [10]. Using a minimum read depth of 4 and read frequency higher than 5 %, little genetic diversity was observed in the relapse cases. However any heterogeneous positions identified were discarded as mapping errors and no validation by Sanger sequencing was done. This may have led to an underestimation of the number of variants, a possibility highlighted by our findings that heterogeneous variants occurring between 30 and 70 % frequency are likely to be truly present within a population.

Our findings which showed the extent of diversity between parental isolates and their respective single colonies are in agreement with a recent study where *in vitro* generated mutants were compared to their drug susceptible progenitor [19]. Numerous variants only present in a proportion of the Illumina reads were identified to be unique to the parental genome. Similar to our findings the authors showed that in some daughter cells the mutant allele was lost while in others it was retained [19]. This suggests that single colonies may not be a true reflection of the genomic diversity of a clinical *M. tuberculosis* isolate. In contrast, WGS of an entire population may underestimate the extent of genetic diversity present in a clinical isolate given the complexity of the *M. tuberculosis* population structure. However, improved read depths may allow for identification of underlying populations which were undetectable in this study. The high degree of genetic diversity seen in this study is similar to that reported elsewhere [5, 12, 14] and is unlikely to have arisen as a consequence of laboratory adaptations. The number of culturing steps was kept to a minimum and previous WGS data from *in vitro* generated mutants in our laboratory showed very little genetic diversity (data not shown). This is supported by WGS of six *M. tuberculosis* H37Rv strains from multiple laboratories that showed little change after years of repeated sub-culturing, suggesting genomic stability during *in vitro* culture [20].

Our results show that single colonies may not be a true reflection of the genetic diversity present within a clinical isolate and vice versa. This finding may have important implications for genomic epidemiology since a recent study by Didelot et al*.* (2014) demonstrated the use of WGS to infer person to person transmission. Underlying populations masked by dominant variants may not truly be represented in the above method and may therefore be overlooked when interpreting person to person transmission [21]. Therefore, this study highlights the importance of the methods of storage used for *M. tuberculosis* isolates. The diversity seen i) within a clinical isolate and ii) between single colonies isolated from a single *M. tuberculosis* clinical isolate needs to be taken into consideration. Storage of samples should therefore be carefully considered based on the research questions which may be asked in future studies. Numerous studies have stated that single colonies were isolated from LJ slants for storage and further use [9, 22–25], while other studies use clinical isolates as a whole representative population [5, 8, 10, 11, 13, 14]. These different approaches may impact results obtained and conclusions drawn.

Having shown genetic diversity between parental populations and their single colonies, as well as diversity between single colonies isolated from the same progenitor, we next investigated the evolution of isoniazid resistance in *M. tuberculosis* clinical isolates within two patients. Initial investigation into genetic heterogeneity in both rifampicin mono-resistant and MDR isolates relative to *M. tuberculosis* H37Rv revealed that there were numerous heterogeneous variants present within the genome. For patient 1, the rifampicin mono-resistant isolate harboured numerous heterogeneous variants, all of which were unique to this isolate when compared to its paired MDR isolate. During the acquisition of the *katG* isoniazid resistance causing mutation all of these heterogeneous variants were lost from the population. This finding suggested that the isoniazid selective pressure imposed an evolutionary bottleneck, resulting in a purification effect and the loss of heterogeneous variants. Contrasting results were observed for patient 2, as the 7 apparently fixed SNPs were lost during the acquisition of the *inhA* promoter isoniazid resistance causing mutation. Once again, this finding suggests that isoniazid selective pressure imposed an evolutionary bottleneck. The paired MDR isolate, R1210, showed a total of 8 heterogeneous variants to be unique to this isolate when compared to R912, two of which were fixed within the population of R912. This suggests that these two variants were reverting to wild type, while other variants were emerging within the population. These findings highlight the continuous genome evolution occurring after an evolutionary bottleneck is imposed on a population. The *M. tuberculosis* isolates from each patient represent two different strain lineages, namely Beijing and EAI, suggesting that genetic diversity observed during the evolution of isoniazid resistance is not limited to one lineage. The MDR isolates were collected approximately 7 and 11 months after the initial rifampicin mono-resistant samples were collected from patients’ 1 and 2, respectively. Unfortunately, clinical information such as treatment regimens and treatment adherence was not available to us at the time of the study, limiting our ability to draw conclusions regarding mutation rates and selective pressure.

The loss of an *rpoC* polymorphism from the rifampicin mono-resistant isolate from patient 1 (R721) further highlights the importance of selective pressure on defining the genetic population of *M. tuberculosis*. A proportion of 30 % of the R721 population (based on read frequency) contained an *rpoC* mutation while there was no *rpoC* mutation present in the follow-up MDR isolate (R807). While variants in the *rpoC* gene have been speculated to be putative compensatory mutations [26], this mutation may not be important for survival since the selective purification for isoniazid resistance causing mutations resulted in the loss of this putative compensatory mutation. No additional compensatory mutations were identified in the WGS data for the isolates used in this study.

Complementary to our findings are the results observed in a recent study by Eldholm et al. where it was shown that the amount of variation between serial isolates from a single patient may be higher than that observed between two patients in a transmission chain [14]. In addition they observed numerous SNPs within the mycobacterial population which occurred concurrently with drug resistance causing mutations, which were termed ‘hitchhiking SNPs’. Excluding these ‘hitchhiking SNPs’ from their analyses, the amount of variation between sequential samples decreased, suggesting that the selective pressure of drug exposure resulted in a purifying effect. Based on these observations the authors concluded that the presence of populations with high genetic diversity (variation) facilitated the emergence of drug resistance, and that selective pressure may be a driving force in longitudinal genetic diversity [14]. Similarly, a recent study by Bergval et al. hypothesised that a selection event may result in the fixation of either a wild-type or mutant allele which was originally present in only a sub-population of *M. tuberculosis* isolates [19].

**Fig. 4:**
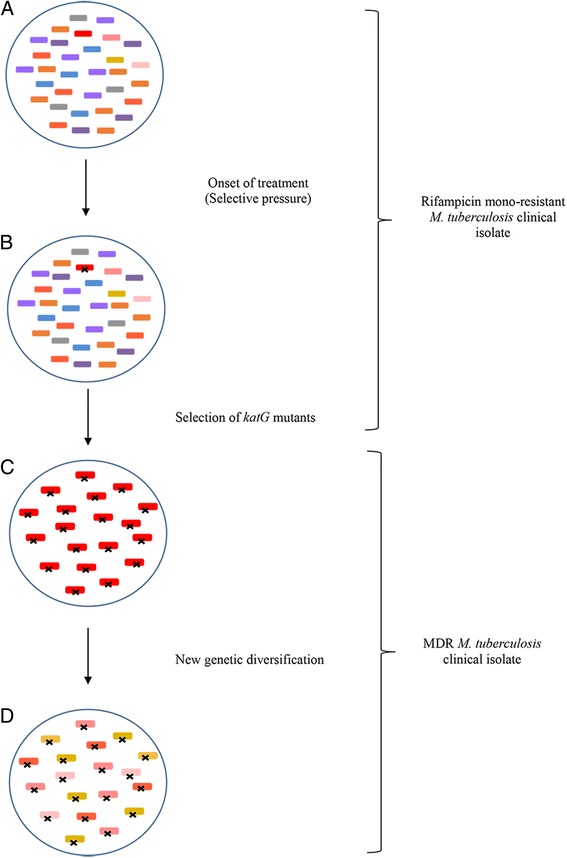
Proposed model for the effect of a selection bottleneck and random mutations on the population structure of *M. tuberculosis* clinical isolates. **a** A rifampicin mono-resistant clinical *M. tuberculosis* isolate where each cell comprising the population contains an *rpoB* mutation. Numerous other genetic mutations are present thereby creating a diverse population structure. (**b**) Following the onset of treatment the genetic mutations in the population may change, and a spontaneous isoniazid resistance causing (for example *katG* gene or *inhA* promoter) mutation is selected for and becomes dominant within the population. (**c**) Selective pressure of treatment results in the emergence of an isoniazid resistant *M. tuberculosis* population where each cell contains a *katG* mutation. Numerous other genetic mutations are lost during the selection bottleneck resulting in a loss of genetic diversity. (**d**) Subsequent replication cycles and population growth results in new genetic mutations arising within the population allowing for new diversification e.g. R1210. Each cell in this MDR population retains the *rpoB* and *katG* resistance causing mutations. Key: x denotes an isoniazid resistance causing mutation (*katG*)

Based on our findings and others’ work, we propose a model to explain the effect of a selection bottleneck and random mutations on the population structure of *M. tuberculosis* clinical isolates (Fig. 4). We hypothesise that during the course of infection numerous genetic mutations arise within a mycobacterial population and in response to a selective pressure such as antibiotic exposure, clones with pre-existing drug resistant mutations are selected. Cells within the population harbouring drug resistant causing mutations survive while drug sensitive cells die. During this process numerous genetic mutations are lost from the population while mutations which occur concurrently with drug resistance causing mutations (or ‘hitchhiking SNPs’) remain [14]. The presence of a selective pressure therefore creates a selection bottleneck, altering the level of genetic diversity within the mycobacterial population. Subsequent growth and the emergence of new SNPs allow for an increase in genetic diversity once again (Fig. 4). This subsequent increase in genetic diversity post selective pressure is substantiated by our findings that a rifampicin mono-resistant isolate and its paired MDR isolate each have numerous unique genetic differences i.e. polymorphisms are lost from the original rifampicin mono-resistant population, while different polymorphisms emerge in the MDR isolate.

## Conclusions

This study investigated two key aspects involved in the use of next-generation WGS. Firstly, we investigated the confidence in the validity of variants called during bioinformatics analysis. Secondly, we investigated the difference in outputs when utilising a relaxed variant filtering approach compared to the standard filtering approach.

To our knowledge this is the first study that has investigated *M. tuberculosis* genomic diversity using single colonies isolated from clinical isolates. The surprisingly high rate of genetic diversity seen in our study is in concordance with previous studies that used a relaxed variant filtering approaches [5, 12, 14]. During the evolution of drug resistance we observed the emergence and disappearance of numerous variants within a population. Our findings allowed us to formulate a model for the selective bottleneck which occurs during the course of infection, acting as a genomic purification event. Subsequent post-bottleneck mycobacterial growth allows for new genetic diversification to occur. This proposed increase in diversity suggests that the genome is preparing to respond to a changing environment.

## Methods

### Strain selection and culture

This study was approved by the Health Research Ethics Committee of Stellenbosch University with the waiver of consent to retrospectively collect routine clinical isolates of *M. tuberculosis* and limited demographic and diagnostic data. To ensure patient confidentiality all patient identifiers were removed. Primary rifampicin mono-resistant clinical *M. tuberculosis* isolates were available from 13 patients that were selected from an extensive longitudinal collection of drug resistant *M. tuberculosis* isolates collected in the Western Cape, South Africa. Two of these patients had follow up isolates which were shown to be multi-drug resistant (MDR) by routine drug susceptibility testing (DST). Each of the 15 isolates were subjected to isoniazid and rifampicin DST [27], and Sanger sequencing of the *inhA* promoter and *katG* and *rpoB* genes (Additional file 2) to confirm the resistance phenotype. In addition, each isolate was further genotyped by spoligotyping and IS*6110* DNA fingerprinting using internationally standardized techniques [28, 29].

BACTEC™ Mycobacterial Growth Indicator tubes (MGIT™ 960) supplemented with Oleic acid-Albumin-Dextrose-Catalase (OADC) were inoculated with each isolate and incubated in the BACTEC™ MGIT™ 960 instrument at 37 °C. Following an indication of growth positivity i.e. when a growth unit of 400 was reached, each MGIT was incubated at 37 °C for an additional 5 days to allow for optimal mycobacterial growth. A volume of 500 μl of positive culture was then used to inoculate a starter culture of 10 ml of 7H9 Middlebrook medium (Becton, Dickinson Microbiology system, Sparks, USA), supplemented with 10 % albumin-dextrose-catalase (ADC), 0.2 % (v/v) glycerol (Merck Laboratories, Saarchem, Gauteng, SA) and 0.1 % Tween80 (Becton, Microbiology systems, Sparks, USA). Subsequently, the starter cultures were grown in filtered screw cap tissue culture flasks (Greiner Bio-one, Maybach Street, Germany) without shaking at 37 °C until an optical density (OD_600_) of 0.6–0.8 was reached. Contamination was assessed by Ziehl-Neelsen (ZN) staining and the plating of cultures onto blood agar plates. A 100 μl aliquot of each starter culture for the rifampicin mono-resistant (*n* = 13) and paired MDR *M. tuberculosis* isolates (*n* = 2) was plated on 7H10 solid media supplemented with OADC for DNA extraction. Serial dilutions were prepared and plated on 7H10 solid media supplemented with OADC and 2 μg/ml rifampicin for selection of single colonies.

### Selection of single colonies

Single colonies were randomly selected from solid media (7H10 Middlebrook media supplemented with OADC) containing 2 μg/ml into Middlebrook 7H9 media supplemented with ADC and 0.1 % Tween80, and statically cultured to an OD_600_ of above 0.8. Each single colony culture was then sub-cultured on solid media (7H10 Middlebrook media supplemented with OADC) for DNA extraction. Unnecessary sub-culturing steps were avoided to minimize the appearance of *in vitro* adaptive mutations.

### DNA extraction and whole genome sequencing

Genomic DNA was isolated from single colonies for each rifampicin mono-resistant *M. tuberculosis* isolate according to standard protocols [30]. In addition, DNA was isolated from the primary cultures of the paired *M. tuberculosis* isolates demonstrating intra-patient evolution of isoniazid resistance i.e. the parental rifampicin mono-resistant isolate and a follow-up MDR isolate. Sequencing libraries for all isolates were constructed using the standard genomic DNA sample preparation kits from Illumina (Illumina, Inc, San Diego, CA), according to manufacturer’s instructions. The whole genomes of the *M. tuberculosis* isolates were sequenced using either the Illumina MiSeq or Illumina HiSeq platforms.

### Mapping and variant detection

An in-house automated pipeline for *M. tuberculosis* next generation sequencing (NGS) analysis was adapted to allow analysis of the sequencing data (Van der Merwe et al*.*, manuscript in preparation). The steps involved in the pipeline are described below.

Quality assessment of the sequencing data (in FASTQ format) was done using FASTQC [31], followed by trimming of adapters and low-quality bases with a Phred quality score of less than 20 and filtering for a minimum read length of 36 using Trimmomatic [32]. A minimum read length of 36 base pairs was used for subsequent mapping. Reads were then mapped to the *M. tuberculosis* H37Rv genome (Genbank: AL123456) using three different mappers namely the Burrows-Wheeler Alignment Tool (BWA) [33], Novoalign [34] and SMALT [35]. For all libraries sequenced, over 98 % of the reference genome was covered by at least one read and an average depth of coverage of 137× was achieved. The alignment files were subjected to local realignment and de-duplication using the Genome Analysis Toolkit (GATK) [36] and Picard [37]. Variants (Single nucleotide polymorphisms (SNPs) and Insertion/Deletions (In/Dels)) in coding as well as non-coding regions were then called from each alignment file using GATK [36], and the overlap of variants identified from all three alignment files was used for further analysis. Variants were annotated using annotation data from Tuberculist [38]. Variants in repetitive regions, such as *pe/ppe* and *pe_pgrs* gene families, were removed from subsequent analysis.

In this study we made use of a relaxed variant filtering approach to allow identification of variants occurring at varying read frequencies i.e. variants present at varying proportions within the population. Variants detected by GATK were filtered for a minimum read depth of 50. An initial read frequency was not defined to allow for the validation of varying frequencies with Sanger sequencing.

Identified variants were compared between the appropriate single colonies selected from the same clinical isolate. Variants present at a frequency ranging from 20–100 % were selected for validation with Sanger sequencing. Variants selected had a minimum read depth of 50 and a mapping quality score of above 50. All variants identified in this study were manually inspected using GenomeView [39].

### Confirmation of variants with Sanger sequencing

A subset of 46 randomly selected genomic variants with read frequencies ranging from 20 to 100 % were validated by targeted PCR and Sanger sequencing. Oligonucleotide primers (Additional file 2) were designed using Primer3 [40] to amplify 300–600 base pairs regions flanking the variant of interest. Briefly, an aliquot of genomic DNA was added to the following reaction mix containing: 1×Q buffer, 1× PCR buffer, 2 mM MgCl_2_, 0.4 mM dNTPs, 50 μM of each primer (Additional file 2) and 1.25U Hot Star Taq polymerase (Qiagen, San Diego, CA, USA). Amplification was done under the following thermocycling conditions: 15 min (min) denaturation at 95 °C followed by 40 amplification cycles (each cycle: 94 °C for 1 min, 62 °C for 1 min, 1 min extension at 72 °C) and an elongation step of 10 min at 72 °C. PCR products were purified and sequenced with the ABI PRISM DNA Sequencer model 377, Perkin Elmer. Sequence polymorphisms were identified by comparing the consensus sequence of each isolate to the corresponding gene sequence of the *M. tuberculosis* H37Rv genome using BioEdit (v7.1.3) [41].

Sequencing results were first inspected for the presence of true variants using the ClustalW multiple alignment tool in the BioEdit software. Furthermore, the chromatograms of each sequencing file were inspected for the presence of both a wild-type and mutant peak to identify heterogeneous variants present at a low percentage within the population. Chromatograms were visually inspected for the presence of mixed peaks at the variant position identified by WGS.

To define a cut-off for the reliability of WGS results for heterogeneous variants a receiver operating characteristic (ROC) analysis was performed using IBM SPSS Statistics version 22 (IBM Corp 2013) [42].

### Availability of supporting data

The data sets supporting the results of this article are available in the European Nucleotide Archive with the following accession number: PRJEB9976 and are available at: http://www.ebi.ac.uk/ena/data/view/PRJEB9976.

The data sets supporting the results for this article are included within the article (and its additional files). Additional file 2(.xls) (Primers used for PCR amplification and Sanger sequencing) is a table listing all primers used in the study to validate variants. Additional file 1 (.xls) (Unique variants identified in the single colony comparison analysis) contains tables of variants identified in the comparison analysis of the genomes of the single colonies isolated from clinical specimens.

## Additional files

Additional file 1:
**Unique variants identified in the single colony comparison analysis.** (XLSX 45 kb) Additional file 2:
**Primers used for PCR amplification and Sanger sequencing.** (XLSX 15 kb)

## Abbreviations

ADC: Albumin Dextrose Catalase
AUC: Area Under the Curve
BWA: Burrow Wheels Aligner
DNA: Deoxyribonucleic Acid
dNTPs: Deoxyribonucleotide Triphosphate
DST: Drug Susceptibility Testing
EAI: East Africa Indian
GATK: Genome Analysis ToolKit
In/Dels: Insertions/Deletions
LAM: Latin American Mediteranean
LCC: Low Copy Clade
MgCl_2_: Magnesium Chloride
MGIT: Mycobacterial Growth Indicator Units
MDR: Multi-drug Resistant
NGS: Next generation sequencing
OADC: Oleic acid albumin dextrose catalase
OD: Optical Density
PCR: Polymerase Chain Reaction
ROC: Receiver operating characteristic
SNPs: Single Nucleotide Polymorphisms
TB: Tuberculosis
WGS: Whole Genome Sequencing
XDR: Extensively Drug Resistant
ZN: Ziehl-Neelsen
